# Chlorido{6,6′-dimethyl-2,2′-[1,2-phenyl­enebis(nitrilo­methyl­idyne)]diphenolato-κ^4^ *O*,*N*,*N*′,*O*′}manganese(III) mono­hydrate

**DOI:** 10.1107/S160053680800620X

**Published:** 2008-03-12

**Authors:** Naser Eltaher Eltayeb, Siang Guan Teoh, Suchada Chantrapromma, Hoong-Kun Fun, Rohana Adnan

**Affiliations:** a School of Chemical Science, Universiti Sains Malaysia, 11800 USM, Penang, Malaysia; b Department of Chemistry, Faculty of Science, Prince of Songkla University, Hat-Yai, Songkhla 90112, Thailand; c X-ray Crystallography Unit, School of Physics, Universiti Sains Malaysia, 11800 USM, Penang, Malaysia

## Abstract

In the title complex, [Mn(C_22_H_18_N_2_O_2_)Cl]·H_2_O, the Mn^III^ center is in a distorted square-pyramidal configuration, with the N_2_O_2_ dianionic tetra­dentate Schiff base ligand in the basal plane and the chloride ion in the apical position. The dihedral angle between the two outer phenolate rings of the tetra­dentate ligand is 8.25 (8)°. The central benzene ring makes dihedral angles of 4.31 (8) and 7.37 (8)° with the two outer phenolate rings. The water mol­ecule links to the complex *via* an O—H⋯Cl hydrogen bond. In addition, in the crystal structure, weak C—H⋯O inter­actions link the mol­ecules into infinite one-dimensional chains along [010]. The crystal is further stabilized by O—H⋯O and O—H⋯Cl hydrogen bonds, together with weak C—H⋯π inter­actions

## Related literature

For bond-length data, see: Allen *et al.* (1987 ▶). For details of ring conformations, see: Cremer & Pople (1975 ▶). For related structures, see for example: Eltayeb *et al.* (2007 ▶); Habibi *et al.* (2007 ▶); Mitra *et al.* (2006 ▶); Naskar *et al.* (2004 ▶). For background to the application of manganese complexes, see for example: Dixit & Srinivasan (1988 ▶); Glatzel *et al.* (2004 ▶); Lu *et al.* (2006 ▶); Stallings *et al.* (1985 ▶).
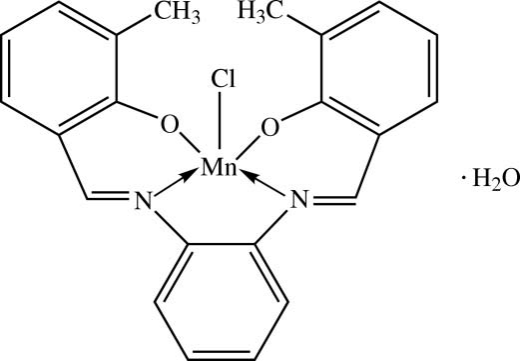


## Experimental

### 

#### Crystal data

[Mn(C_22_H_18_N_2_O_2_)Cl]·H_2_O
*M* *_r_* = 450.79
Monoclinic, 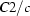

*a* = 27.1836 (6) Å
*b* = 6.8033 (1) Å
*c* = 21.8896 (4) Å
β = 108.976 (1)°
*V* = 3828.22 (12) Å^3^
*Z* = 8
Mo *K*α radiation
μ = 0.86 mm^−1^
*T* = 100.0 (1) K
0.42 × 0.26 × 0.11 mm

#### Data collection

Bruker SMART APEX2 CCD area-detector diffractometer
Absorption correction: multi-scan (*SADABS*; Bruker, 2005 ▶) *T*_min_ = 0.714, *T*_max_ = 0.915
24765 measured reflections
5586 independent reflections
4459 reflections with *I* > 2σ(*I*)
*R*_int_ = 0.038

#### Refinement

*R*[*F* ^2^ > 2σ(*F* ^2^)] = 0.037
*wR*(*F* ^2^) = 0.100
*S* = 1.10
5586 reflections
264 parameters
H-atom parameters constrained
Δρ_max_ = 0.49 e Å^−3^
Δρ_min_ = −0.39 e Å^−3^

### 

Data collection: *APEX2* (Bruker, 2005 ▶); cell refinement: *APEX2*; data reduction: *SAINT* (Bruker, 2005 ▶); program(s) used to solve structure: *SHELXTL* (Sheldrick, 2008 ▶); program(s) used to refine structure: *SHELXTL*; molecular graphics: *SHELXTL*; software used to prepare material for publication: *SHELXTL* and *PLATON* (Spek, 2003 ▶).

## Supplementary Material

Crystal structure: contains datablocks global, I. DOI: 10.1107/S160053680800620X/sj2469sup1.cif

Structure factors: contains datablocks I. DOI: 10.1107/S160053680800620X/sj2469Isup2.hkl

Additional supplementary materials:  crystallographic information; 3D view; checkCIF report

## Figures and Tables

**Table 1:** Hydrogen-bond geometry (Å, °)

*D*—H⋯*A*	*D*—H	H⋯*A*	*D*⋯*A*	*D*—H⋯*A*
O1*W*—H1*W*1⋯Cl1	0.87	2.54	3.3544 (16)	157
O1*W*—H2*W*1⋯O1^i^	0.84	2.43	3.191 (2)	151
O1*W*—H2*W*1⋯O2^i^	0.84	2.53	3.2642 (19)	146
C16—H16*A*⋯O1*W*^ii^	0.93	2.48	3.364 (2)	160
C7—H7*A*⋯*Cg*1^iii^	0.93	3.39	3.9811 (17)	123

Symmetry codes: (i) 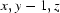; (ii) 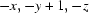; (iii) 
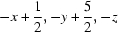
. *Cg*1 is the centroid of the C1–C6 benzene ring.

**Fig. 1.**
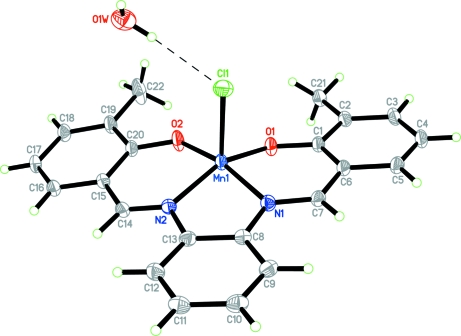
The asymmetric unit of (I), showing 50% probability displacement ellipsoids and the atomic numbering. The O—H···Cl hydrogen bond is drawn as a dashed line

**Fig. 2.**
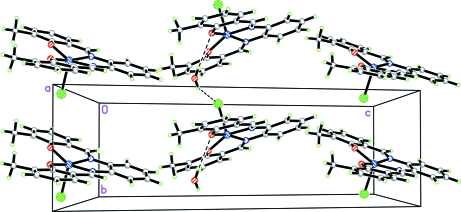
The crystal packing of (I), viewed along the a axis showing the chains running along the [0 1 0] direction. Hydrogen bonds are drawn as dashed lines.

## supplementary crystallographic information

### Comment

Schiff base ligands containing strong donor sites such as oxygen and imine nitrogen atoms and their metal complexes have been the subject of extensive investigation. Manganese complexes with Schiff base ligands have attracted considerable interest in the past decades and recently, due to their variety of applications in chemistry, biology, physics and advanced materials. They have been used as models for the oxygen-evolving complex of photosystem II (Glatzel *et al.*, 2004), in catalysis (Dixit and Srinivasan, 1988), as single-molecule magnets (Lu *et al.*, 2006) and serve as models for the active sites of manganese-containing metal enzymes (Stallings *et al.*, 1985). Recently, we reported the crystal structure of 4,4'-Dimethoxy-2,2'-[1,2-phenylenebis(nitrilomethylidyne)]diphenol, (Eltayeb *et al.*, 2007). We report here the crystal structure of a Mn(III) complex of the closely related ligand 2,2'-{1,2-phenylenebis[nitrilomethylylidene]}bis(6-methylphenol).

In the title complex molecule (Fig. 1), the coordination sphere of the Mn^III^ ion is a slightly distorted square-pyramid consisting of the N_2_O_2_ coordination plane of the dianionic tetradentate Schiff base ligand (coordinating through N1, N2, O1 and O2) and the axially bound chloride ion. The Mn—O distances [Mn1—O1 = 1.8672 (11) Å and Mn1—O2 = 1.8587 (13) Å] and Mn—N distances [Mn1—N1 = 1.9887 (4) Å and Mn1—N2 = 1.9922 (13) Å are quite similar to those observed in other related Mn^III^ complexes of N_2_O_2_ Schiff base ligands (Habibi *et al.*, 2007; Mitra *et al.*, 2006). Other bond lengths and angles observed in the structure are also normal (Allen *et al.*, 1987). Coordination of the the N_2_O_2_ chelate ligand to the Mn^III^ ion results in the formation of a planar five-membered ring (Mn1/N1/N2/C8/C13) and two six-membered rings; the Mn1/O2/N2/C14/C15/C20 ring is almost planar whereas the Mn1/O1/N1/C1/C6/C7 ring adopts an envelope conformation with atom O1 displaced from the Mn1/N1/C1/C6/C7 plane by 0.159 (1) Å and with Cremer & Pople (1975) puckering parameters Q = 0.274 (1) °, θ = 61.3 (4) ° and φ = 12.8 (4) °. The dihedral angle between the two outer phenolate rings [C1–C6 and C15–C20] of the Schiff base ligand is 8.25 (8) °. The central benzene ring (C8–C13) makes dihedral angles of 4.31 (8) ° and 7.37 (8) ° with the two outer phenolate rings, respectively. The water molecule forms an O—H···Cl hydrogen bond with the complex.

In the crystal packing (Fig. 2), a weak C—H···O interaction [C16—H16···O1W; symmetry code -*x*, 1 - *y*, -*z* (Table 1)] links the molecules into infinite one-dimensional chains along the [0 1 0] direction. The crystal is further stabilized by O—H···O and O—H···Cl hydrogen bonds, together with weak C—H···π interactions (Table 1); *Cg*_1_ is the centroid of the C1–C6 benzene ring.

### Experimental

The title compound was synthesized by adding 2-hydroxy-3-methylbenzaldehyde (0.5 ml, 4 mmol) to a solution of *o*-phenylenediamine (0.216 g, 2 mmol) in ethanol 95% (30 ml). The mixture was refluxed with stirring for half an hour. Manganese chloride tetrahydrate (0.394 g, 2 mmol) in ethanol (10 ml) was then added, followed by triethylamine (0.5 ml, 3.6 mmol). The mixture was refluxed at room temperature for three hours. A brown precipitate was obtained, washed with about 5 ml e thanol, dried, and then washed with copious quantities of diethylether. Brown single crystals of the title compound suitable for *x*-ray structure determination were recrystallized from methanol by slow evaporation of the solvent at room temperature over several days.

### Refinement

All H atoms were placed in calculated positions with d(O—H) = 0.84 and 0.87 Å, *U*_iso_=1.2*U*_eq_, d(C—H) = 0.93 Å, *U*_iso_=1.2*U*_eq_(C) for aromatic, 0.98 Å, *U*_iso_ = 1.2*U*_eq_(C) for CH, 0.96 Å, *U*_iso_ = 1.5*U*_eq_(C) for CH_3_ atoms. A rotating group model was used for the methyl groups. The highest residual electron density peak is located at 0.70 Å from C15 and the deepest hole is located at 0.66 Å from Mn1.

### Crystal data

**Table:**

[Mn(C_22_H_18_N_2_O_2_)Cl]·H_2_O	*F*_000_ = 1856
*M**_r_* = 450.79	*D*_x_ = 1.564 Mg m^−^^3^
Monoclinic, *C*2/*c*	Mo *K*α radiation λ = 0.71073 Å
Hall symbol: -C 2yc	Cell parameters from 5586 reflections
*a* = 27.1836 (6) Å	θ = 2.1–30.0º
*b* = 6.8033 (1) Å	µ = 0.86 mm^−^^1^
*c* = 21.8896 (4) Å	*T* = 100.0 (1) K
β = 108.976 (1)º	Block, brown
*V* = 3828.22 (12) Å^3^	0.42 × 0.26 × 0.11 mm
*Z* = 8	

### Data collection

**Table:**

Bruker SMART APEX2 CCD area-detector diffractometer	5586 independent reflections
Radiation source: fine-focus sealed tube	4459 reflections with *I* > 2σ(*I*)
Monochromator: graphite	*R*_int_ = 0.038
Detector resolution: 8.33 pixels mm^-1^	θ_max_ = 30.0º
*T* = 100.0(1) K	θ_min_ = 2.1º
ω scans	*h* = −38→38
Absorption correction: multi-scan(SADABS; Bruker, 2005)	*k* = −9→9
*T*_min_ = 0.714, *T*_max_ = 0.915	*l* = −30→30
24765 measured reflections	

### Refinement

**Table:**

Refinement on *F*^2^	Secondary atom site location: difference Fourier map
Least-squares matrix: full	Hydrogen site location: inferred from neighbouring sites
*R*[*F*^2^ > 2σ(*F*^2^)] = 0.037	H-atom parameters constrained
*wR*(*F*^2^) = 0.100	*w* = 1/[σ^2^(*F*_o_^2^) + (0.049*P*)^2^ + 1.3516*P*] where *P* = (*F*_o_^2^ + 2*F*_c_^2^)/3
*S* = 1.10	(Δ/σ)_max_ < 0.001
5586 reflections	Δρ_max_ = 0.49 e Å^−^^3^
264 parameters	Δρ_min_ = −0.38 e Å^−^^3^
Primary atom site location: structure-invariant direct methods	Extinction correction: none

### Special details

**Table:**

Experimental. The low-temperature data was collected with the Oxford Cyrosystem Cobra low-temperature attachment.
Geometry. All e.s.d.'s (except the e.s.d. in the dihedral angle between two l.s. planes) are estimated using the full covariance matrix. The cell e.s.d.'s are taken into account individually in the estimation of e.s.d.'s in distances, angles and torsion angles; correlations between e.s.d.'s in cell parameters are only used when they are defined by crystal symmetry. An approximate (isotropic) treatment of cell e.s.d.'s is used for estimating e.s.d.'s involving l.s. planes.
Refinement. Refinement of *F*^2^ against ALL reflections. The weighted *R*-factor *wR* and goodness of fit *S* are based on *F*^2^, conventional *R*-factors *R* are based on *F*, with *F* set to zero for negative *F*^2^. The threshold expression of *F*^2^ > σ(*F*^2^) is used only for calculating *R*-factors(gt) *etc*. and is not relevant to the choice of reflections for refinement. *R*-factors based on *F*^2^ are statistically about twice as large as those based on *F*, and *R*- factors based on ALL data will be even larger.

### Fractional atomic coordinates and isotropic or equivalent isotropic displacement parameters (Å^2^)

**Table:**

	*x*	*y*	*z*	*U*_iso_*/*U*_eq_	
Mn1	0.132825 (10)	0.85182 (4)	0.049972 (11)	0.01683 (8)	
Cl1	0.174472 (18)	0.53896 (6)	0.07957 (2)	0.02546 (11)	
O1	0.17748 (5)	1.01562 (18)	0.11197 (5)	0.0196 (3)	
O2	0.08842 (5)	0.8545 (2)	0.09903 (6)	0.0257 (3)	
N1	0.16961 (6)	0.9072 (2)	−0.01268 (6)	0.0174 (3)	
N2	0.07481 (6)	0.7877 (2)	−0.03041 (6)	0.0169 (3)	
C1	0.22593 (6)	1.0630 (2)	0.11779 (8)	0.0177 (3)	
C2	0.25769 (7)	1.1380 (2)	0.17830 (8)	0.0185 (3)	
C3	0.30824 (7)	1.1903 (3)	0.18499 (8)	0.0216 (4)	
H3A	0.3294	1.2374	0.2247	0.026*	
C4	0.32889 (7)	1.1756 (3)	0.13454 (9)	0.0226 (4)	
H4A	0.3630	1.2136	0.1408	0.027*	
C5	0.29856 (7)	1.1049 (3)	0.07576 (8)	0.0207 (3)	
H5A	0.3121	1.0960	0.0419	0.025*	
C6	0.24664 (7)	1.0451 (2)	0.06635 (8)	0.0181 (3)	
C7	0.21695 (7)	0.9778 (2)	0.00333 (8)	0.0178 (3)	
H7A	0.2324	0.9846	−0.0288	0.021*	
C8	0.14187 (7)	0.8456 (2)	−0.07694 (8)	0.0175 (3)	
C9	0.16154 (7)	0.8447 (2)	−0.12824 (8)	0.0207 (3)	
H9A	0.1953	0.8873	−0.1223	0.025*	
C10	0.13042 (8)	0.7801 (3)	−0.18802 (8)	0.0237 (4)	
H10A	0.1434	0.7798	−0.2225	0.028*	
C11	0.08000 (8)	0.7153 (3)	−0.19751 (8)	0.0240 (4)	
H11A	0.0596	0.6715	−0.2381	0.029*	
C12	0.05992 (7)	0.7154 (3)	−0.14702 (8)	0.0219 (4)	
H12A	0.0262	0.6722	−0.1535	0.026*	
C13	0.09089 (7)	0.7810 (2)	−0.08629 (7)	0.0178 (3)	
C14	0.02675 (7)	0.7584 (2)	−0.03375 (7)	0.0175 (3)	
H14A	0.0030	0.7334	−0.0744	0.021*	
C15	0.00722 (6)	0.7612 (2)	0.01947 (7)	0.0165 (3)	
C16	−0.04602 (7)	0.7183 (2)	0.00705 (8)	0.0194 (3)	
H16A	−0.0668	0.6882	−0.0348	0.023*	
C17	−0.06737 (7)	0.7204 (3)	0.05540 (8)	0.0209 (3)	
H17A	−0.1025	0.6931	0.0466	0.025*	
C18	−0.03579 (7)	0.7639 (3)	0.11849 (8)	0.0228 (4)	
H18A	−0.0505	0.7646	0.1514	0.027*	
C19	0.01650 (7)	0.8058 (3)	0.13328 (8)	0.0226 (4)	
C20	0.03867 (7)	0.8075 (2)	0.08341 (8)	0.0181 (3)	
C21	0.23506 (7)	1.1618 (3)	0.23208 (8)	0.0228 (4)	
H21A	0.2627	1.1804	0.2722	0.034*	
H21B	0.2156	1.0462	0.2348	0.034*	
H21C	0.2124	1.2741	0.2236	0.034*	
C22	0.05095 (8)	0.8465 (4)	0.20117 (9)	0.0432 (6)	
H22A	0.0299	0.8682	0.2282	0.065*	
H22B	0.0716	0.9612	0.2016	0.065*	
H22C	0.0734	0.7359	0.2171	0.065*	
O1W	0.10173 (6)	0.3038 (2)	0.15433 (6)	0.0365 (4)	
H1W1	0.1210	0.3909	0.1437	0.055*	
H2W1	0.1124	0.1985	0.1426	0.055*	

### Atomic displacement parameters (Å^2^)

**Table:**

	*U*^11^	*U*^22^	*U*^33^	*U*^12^	*U*^13^	*U*^23^
Mn1	0.01223 (13)	0.02391 (14)	0.01463 (12)	−0.00348 (10)	0.00478 (9)	−0.00113 (9)
Cl1	0.0266 (2)	0.0250 (2)	0.0228 (2)	0.00082 (18)	0.00529 (17)	0.00321 (15)
O1	0.0134 (6)	0.0263 (6)	0.0202 (5)	−0.0048 (5)	0.0070 (5)	−0.0047 (5)
O2	0.0138 (6)	0.0470 (8)	0.0169 (5)	−0.0103 (6)	0.0060 (5)	−0.0055 (5)
N1	0.0156 (7)	0.0198 (6)	0.0170 (6)	−0.0003 (6)	0.0056 (5)	−0.0001 (5)
N2	0.0163 (7)	0.0194 (6)	0.0147 (6)	−0.0012 (6)	0.0049 (5)	0.0005 (5)
C1	0.0132 (8)	0.0184 (8)	0.0218 (7)	−0.0005 (6)	0.0061 (6)	0.0010 (6)
C2	0.0143 (8)	0.0195 (8)	0.0215 (7)	−0.0001 (6)	0.0053 (6)	−0.0002 (6)
C3	0.0133 (8)	0.0227 (8)	0.0264 (8)	−0.0020 (7)	0.0030 (7)	−0.0015 (6)
C4	0.0122 (8)	0.0242 (9)	0.0314 (9)	−0.0012 (7)	0.0072 (7)	0.0019 (7)
C5	0.0164 (8)	0.0219 (8)	0.0267 (8)	0.0008 (7)	0.0108 (7)	0.0035 (6)
C6	0.0134 (8)	0.0191 (8)	0.0221 (7)	0.0003 (6)	0.0063 (6)	0.0022 (6)
C7	0.0154 (8)	0.0191 (8)	0.0207 (7)	0.0009 (6)	0.0082 (6)	0.0017 (6)
C8	0.0191 (8)	0.0174 (7)	0.0162 (7)	0.0012 (6)	0.0060 (6)	0.0011 (6)
C9	0.0222 (9)	0.0201 (8)	0.0218 (8)	0.0017 (7)	0.0100 (7)	0.0031 (6)
C10	0.0291 (10)	0.0254 (8)	0.0192 (7)	0.0043 (8)	0.0113 (7)	0.0020 (6)
C11	0.0276 (10)	0.0270 (9)	0.0158 (7)	0.0013 (8)	0.0047 (7)	−0.0002 (6)
C12	0.0213 (9)	0.0254 (8)	0.0181 (7)	−0.0004 (7)	0.0054 (7)	0.0002 (6)
C13	0.0192 (8)	0.0197 (8)	0.0152 (7)	0.0012 (7)	0.0065 (6)	0.0011 (6)
C14	0.0158 (8)	0.0187 (8)	0.0159 (7)	−0.0013 (6)	0.0024 (6)	−0.0010 (6)
C15	0.0138 (8)	0.0174 (7)	0.0178 (7)	−0.0004 (6)	0.0044 (6)	−0.0006 (6)
C16	0.0161 (8)	0.0209 (8)	0.0195 (7)	−0.0023 (7)	0.0032 (6)	−0.0016 (6)
C17	0.0123 (8)	0.0248 (8)	0.0256 (8)	−0.0037 (7)	0.0060 (6)	−0.0015 (7)
C18	0.0153 (9)	0.0325 (9)	0.0221 (8)	−0.0043 (7)	0.0081 (7)	−0.0007 (7)
C19	0.0151 (8)	0.0346 (9)	0.0184 (7)	−0.0055 (7)	0.0061 (6)	−0.0022 (7)
C20	0.0131 (8)	0.0236 (8)	0.0177 (7)	−0.0040 (6)	0.0051 (6)	−0.0011 (6)
C21	0.0173 (9)	0.0272 (9)	0.0232 (8)	−0.0018 (7)	0.0056 (7)	−0.0018 (7)
C22	0.0216 (10)	0.0924 (19)	0.0182 (8)	−0.0194 (11)	0.0100 (8)	−0.0081 (10)
O1W	0.0398 (9)	0.0460 (8)	0.0237 (6)	0.0046 (7)	0.0105 (6)	0.0020 (6)

### Geometric parameters (Å, °)

**Table:**

Mn1—O2	1.8585 (13)	C10—C11	1.389 (3)
Mn1—O1	1.8671 (11)	C10—H10A	0.9300
Mn1—N1	1.9786 (14)	C11—C12	1.383 (3)
Mn1—N2	1.9921 (13)	C11—H11A	0.9300
Mn1—Cl1	2.3989 (5)	C12—C13	1.396 (2)
O1—C1	1.321 (2)	C12—H12A	0.9300
O2—C20	1.322 (2)	C14—C15	1.429 (2)
N1—C7	1.310 (2)	C14—H14A	0.9300
N1—C8	1.426 (2)	C15—C16	1.413 (2)
N2—C14	1.300 (2)	C15—C20	1.418 (2)
N2—C13	1.427 (2)	C16—C17	1.363 (2)
C1—C6	1.418 (2)	C16—H16A	0.9300
C1—C2	1.420 (2)	C17—C18	1.400 (2)
C2—C3	1.380 (2)	C17—H17A	0.9300
C2—C21	1.504 (2)	C18—C19	1.381 (2)
C3—C4	1.396 (3)	C18—H18A	0.9300
C3—H3A	0.9300	C19—C20	1.408 (2)
C4—C5	1.370 (2)	C19—C22	1.502 (2)
C4—H4A	0.9300	C21—H21A	0.9600
C5—C6	1.418 (2)	C21—H21B	0.9600
C5—H5A	0.9300	C21—H21C	0.9600
C6—C7	1.428 (2)	C22—H22A	0.9600
C7—H7A	0.9300	C22—H22B	0.9600
C8—C9	1.392 (2)	C22—H22C	0.9600
C8—C13	1.404 (2)	O1W—H1W1	0.8718
C9—C10	1.379 (2)	O1W—H2W1	0.8438
C9—H9A	0.9300		
			
O2—Mn1—O1	88.00 (5)	C9—C10—H10A	119.5
O2—Mn1—N1	165.54 (6)	C11—C10—H10A	119.5
O1—Mn1—N1	91.97 (5)	C12—C11—C10	120.46 (16)
O2—Mn1—N2	92.07 (6)	C12—C11—H11A	119.8
O1—Mn1—N2	156.00 (6)	C10—C11—H11A	119.8
N1—Mn1—N2	82.12 (6)	C11—C12—C13	119.28 (17)
O2—Mn1—Cl1	101.00 (4)	C11—C12—H12A	120.4
O1—Mn1—Cl1	101.25 (4)	C13—C12—H12A	120.4
N1—Mn1—Cl1	93.19 (4)	C12—C13—C8	120.00 (16)
N2—Mn1—Cl1	102.29 (4)	C12—C13—N2	124.66 (16)
C1—O1—Mn1	127.44 (11)	C8—C13—N2	115.34 (14)
C20—O2—Mn1	130.55 (11)	N2—C14—C15	125.81 (14)
C7—N1—C8	122.01 (15)	N2—C14—H14A	117.1
C7—N1—Mn1	123.94 (11)	C15—C14—H14A	117.1
C8—N1—Mn1	113.79 (11)	C16—C15—C20	119.12 (15)
C14—N2—C13	121.95 (13)	C16—C15—C14	117.97 (14)
C14—N2—Mn1	125.15 (11)	C20—C15—C14	122.91 (15)
C13—N2—Mn1	112.88 (11)	C17—C16—C15	121.10 (15)
O1—C1—C6	122.88 (14)	C17—C16—H16A	119.4
O1—C1—C2	117.53 (15)	C15—C16—H16A	119.4
C6—C1—C2	119.58 (15)	C16—C17—C18	119.26 (16)
C3—C2—C1	118.29 (16)	C16—C17—H17A	120.4
C3—C2—C21	122.38 (15)	C18—C17—H17A	120.4
C1—C2—C21	119.32 (15)	C19—C18—C17	121.95 (17)
C2—C3—C4	122.69 (16)	C19—C18—H18A	119.0
C2—C3—H3A	118.7	C17—C18—H18A	119.0
C4—C3—H3A	118.7	C18—C19—C20	119.16 (15)
C5—C4—C3	119.62 (17)	C18—C19—C22	122.21 (17)
C5—C4—H4A	120.2	C20—C19—C22	118.63 (16)
C3—C4—H4A	120.2	O2—C20—C19	117.46 (14)
C4—C5—C6	120.24 (16)	O2—C20—C15	123.15 (15)
C4—C5—H5A	119.9	C19—C20—C15	119.39 (15)
C6—C5—H5A	119.9	C2—C21—H21A	109.5
C5—C6—C1	119.56 (15)	C2—C21—H21B	109.5
C5—C6—C7	117.09 (15)	H21A—C21—H21B	109.5
C1—C6—C7	123.28 (15)	C2—C21—H21C	109.5
N1—C7—C6	124.99 (15)	H21A—C21—H21C	109.5
N1—C7—H7A	117.5	H21B—C21—H21C	109.5
C6—C7—H7A	117.5	C19—C22—H22A	109.5
C9—C8—C13	120.07 (15)	C19—C22—H22B	109.5
C9—C8—N1	125.34 (16)	H22A—C22—H22B	109.5
C13—C8—N1	114.59 (14)	C19—C22—H22C	109.5
C10—C9—C8	119.27 (17)	H22A—C22—H22C	109.5
C10—C9—H9A	120.4	H22B—C22—H22C	109.5
C8—C9—H9A	120.4	H1W1—O1W—H2W1	101.5
C9—C10—C11	120.92 (17)		
			
O2—Mn1—O1—C1	168.47 (14)	C5—C6—C7—N1	175.99 (16)
N1—Mn1—O1—C1	−26.00 (14)	C1—C6—C7—N1	−7.1 (3)
N2—Mn1—O1—C1	−100.93 (18)	C7—N1—C8—C9	2.8 (3)
Cl1—Mn1—O1—C1	67.65 (13)	Mn1—N1—C8—C9	−171.59 (13)
O1—Mn1—O2—C20	162.44 (16)	C7—N1—C8—C13	−177.59 (15)
N1—Mn1—O2—C20	72.3 (3)	Mn1—N1—C8—C13	8.05 (18)
N2—Mn1—O2—C20	6.45 (16)	C13—C8—C9—C10	0.0 (2)
Cl1—Mn1—O2—C20	−96.49 (15)	N1—C8—C9—C10	179.64 (16)
O2—Mn1—N1—C7	109.0 (2)	C8—C9—C10—C11	−0.3 (3)
O1—Mn1—N1—C7	19.33 (14)	C9—C10—C11—C12	0.3 (3)
N2—Mn1—N1—C7	175.97 (14)	C10—C11—C12—C13	−0.1 (3)
Cl1—Mn1—N1—C7	−82.06 (13)	C11—C12—C13—C8	−0.1 (2)
O2—Mn1—N1—C8	−76.8 (2)	C11—C12—C13—N2	−179.76 (15)
O1—Mn1—N1—C8	−166.43 (11)	C9—C8—C13—C12	0.2 (2)
N2—Mn1—N1—C8	−9.80 (11)	N1—C8—C13—C12	−179.47 (15)
Cl1—Mn1—N1—C8	92.18 (11)	C9—C8—C13—N2	179.84 (14)
O2—Mn1—N2—C14	−2.08 (14)	N1—C8—C13—N2	0.2 (2)
O1—Mn1—N2—C14	−91.82 (19)	C14—N2—C13—C12	−10.0 (3)
N1—Mn1—N2—C14	−168.78 (14)	Mn1—N2—C13—C12	171.41 (13)
Cl1—Mn1—N2—C14	99.64 (13)	C14—N2—C13—C8	170.41 (15)
O2—Mn1—N2—C13	176.49 (11)	Mn1—N2—C13—C8	−8.22 (18)
O1—Mn1—N2—C13	86.76 (17)	C13—N2—C14—C15	179.65 (15)
N1—Mn1—N2—C13	9.79 (11)	Mn1—N2—C14—C15	−1.9 (2)
Cl1—Mn1—N2—C13	−81.78 (11)	N2—C14—C15—C16	−177.54 (16)
Mn1—O1—C1—C6	20.1 (2)	N2—C14—C15—C20	3.3 (3)
Mn1—O1—C1—C2	−161.02 (11)	C20—C15—C16—C17	−0.2 (2)
O1—C1—C2—C3	−179.27 (15)	C14—C15—C16—C17	−179.31 (15)
C6—C1—C2—C3	−0.3 (2)	C15—C16—C17—C18	−0.6 (3)
O1—C1—C2—C21	−0.6 (2)	C16—C17—C18—C19	0.3 (3)
C6—C1—C2—C21	178.32 (15)	C17—C18—C19—C20	0.8 (3)
C1—C2—C3—C4	1.1 (3)	C17—C18—C19—C22	−178.0 (2)
C21—C2—C3—C4	−177.49 (16)	Mn1—O2—C20—C19	173.81 (12)
C2—C3—C4—C5	−0.6 (3)	Mn1—O2—C20—C15	−6.8 (3)
C3—C4—C5—C6	−0.6 (3)	C18—C19—C20—O2	177.84 (17)
C4—C5—C6—C1	1.4 (2)	C22—C19—C20—O2	−3.2 (3)
C4—C5—C6—C7	178.40 (15)	C18—C19—C20—C15	−1.6 (3)
O1—C1—C6—C5	178.01 (15)	C22—C19—C20—C15	177.32 (18)
C2—C1—C6—C5	−0.9 (2)	C16—C15—C20—O2	−178.13 (16)
O1—C1—C6—C7	1.2 (3)	C14—C15—C20—O2	1.0 (3)
C2—C1—C6—C7	−177.73 (15)	C16—C15—C20—C19	1.3 (2)
C8—N1—C7—C6	178.93 (15)	C14—C15—C20—C19	−179.62 (16)
Mn1—N1—C7—C6	−7.3 (2)		

### Hydrogen-bond geometry (Å, °)

**Table:**

*D*—H···*A*	*D*—H	H···*A*	*D*···*A*	*D*—H···*A*
O1W—H1W1···Cl1	0.87	2.54	3.3544 (16)	157
O1W—H2W1···O1^i^	0.84	2.43	3.191 (2)	151
O1W—H2W1···O2^i^	0.84	2.53	3.2642 (19)	146
C16—H16A···O1W^ii^	0.93	2.48	3.364 (2)	160
C7—H7A···Cg1^iii^	0.93	3.39	3.9811 (17)	123

Symmetry codes:  (i) *x*, *y*−1, *z*; (ii) −*x*, −*y*+1, −*z*; (iii) −*x*+1/2, −*y*+5/2, −*z*.
